# Spatial transcriptomics reveals altered lipid metabolism and inflammation-related gene expression of sebaceous glands in psoriasis and atopic dermatitis

**DOI:** 10.3389/fimmu.2024.1334844

**Published:** 2024-02-16

**Authors:** Peter Seiringer, Christina Hillig, Alexander Schäbitz, Manja Jargosch, Anna Caroline Pilz, Stefanie Eyerich, Andrea Szegedi, Michaela Sochorová, Florian Gruber, Christos C. Zouboulis, Tilo Biedermann, Michael P. Menden, Kilian Eyerich, Daniel Törőcsik

**Affiliations:** ^1^ Department of Dermatology and Allergy, Technical University of Munich, Munich, Germany; ^2^ Division of Dermatology and Venereology, Department of Medicine Solna, and Center for Molecular Medicine, Karolinska Institutet, Stockholm, Sweden; ^3^ Institute of Computational Biology, Helmholtz Zentrum München - German Research Centre for Environmental Health, Munich, Germany; ^4^ Zentrum für Allergie und Umwelt (ZAUM) - Center of Allergy and Environment, Technical University of Munich and Helmholtz Zentrum München, Munich, Germany; ^5^ Department of Dermatology and Venereology, Medical Center, University of Freiburg, Freiburg, Germany; ^6^ Department of Dermatology, Faculty of Medicine, University of Debrecen, Debrecen, Hungary; ^7^ Hungarian Research Network (HUN-REN DE), Allergology Research Group, Debrecen, Hungary; ^8^ Division for Biology and Pathobiology of the Skin, Department of Dermatology, Medical University of Vienna, Vienna, Austria; ^9^ Christian Doppler Laboratory for Skin Multimodal Analytical Imaging of Aging and Senescence (SKINMAGINE), Medical University of Vienna, Vienna, Austria; ^10^ Departments of Dermatology, Venereology, Allergology and Immunology, Staedtisches Klinikum Dessau, Brandenburg Medical School Theodor Fontane and Faculty of Health Sciences Brandenburg, Dessau, Germany; ^11^ Department of Biochemistry and Pharmacology, University of Melbourne, Melbourne, VIC, Australia

**Keywords:** sebaceous glands, psoriasis, atopic dermatitis (AD), spatial transcriptomics, lipid metabolism, inflammatory skin diseases

## Abstract

Sebaceous glands drive acne, however, their role in other inflammatory skin diseases remains unclear. To shed light on their potential contribution to disease development, we investigated the spatial transcriptome of sebaceous glands in psoriasis and atopic dermatitis patients across lesional and non-lesional human skin samples. Both atopic dermatitis and psoriasis sebaceous glands expressed genes encoding key proteins for lipid metabolism and transport such as *ALOX15B, APOC1, FABP7, FADS1/2, FASN, PPARG*, and *RARRES1.* Also, inflammation-related *SAA1* was identified as a common spatially variable gene. In atopic dermatitis, genes mainly related to lipid metabolism (e.g. *ACAD8, FADS6*, or *EBP)* as well as disease-specific genes, i.e., Th2 inflammation-related lipid-regulating *HSD3B1* were differentially expressed. On the contrary, in psoriasis, more inflammation-related spatially variable genes (e.g. *SERPINF1*, *FKBP5*, *IFIT1/3, DDX58*) were identified. Other psoriasis-specific enriched pathways included lipid metabolism (e.g. *ACOT4, S1PR3)*, keratinization (e.g. *LCE5A, KRT5/7/16*), neutrophil degranulation, and antimicrobial peptides (e.g. *LTF, DEFB4A, S100A7-9*). In conclusion, our results show that sebaceous glands contribute to skin homeostasis with a cell type-specific lipid metabolism, which is influenced by the inflammatory microenvironment. These findings further support that sebaceous glands are not bystanders in inflammatory skin diseases, but can actively and differentially modulate inflammation in a disease-specific manner.

## Introduction

Acne, one of the most prevalent diseases in adolescents, provides evidence that sebocytes may be disease drivers by increasing lipid production (1–4). Gene expression analyses of whole tissue acne samples and sebocyte cell lines showed that sebocytes are able to respond to a wide repertoire of both local and systemic stimuli, such as hormones, growth factors and neuroendocrine mediators, with an increased expression of inflammatory cytokines, cholesterol biosynthesis, cyclooxygenase and lipoxygenase (5, 6). This suggests that sebocytes may contribute to the pathogenesis of acne and have a complex impact on skin metabolism and inflammation. Advances in sebaceous gland (SG) research including the detection of Toll-like receptors (TLRs) on the surface of SGs (7), changes in gene expression patterns in response to their activation (8, 9), and the production of antimicrobial peptides (10–13) have led to the introduction of “sebaceous-immunobiology” (14), suggesting that the active role of SGs in disease pathogenesis may extend far beyond acne.

Results from immunostainings and whole tissue gene expression data suggest that seborrhoeic dermatitis is centered around dysfunctional SGs, in which metabolized sebum lipids may induce inflammation (15, 16). The presence of enlarged SGs in rosacea also suggests a central role in the pathology of this disease (17, 18). Therefore, SG-rich areas, enlarged SGs and seborrhoea are thought to contribute to inflammatory skin diseases. However, our increasing knowledge of the immune-competence of sebocytes allowed further intriguing speculations as to whether SGs could indeed independently drive disease pathologies in two of the major inflammatory skin diseases such as atopic dermatitis (AD) and psoriasis (PSO).

AD is characterized by dry skin and inflammation, starting in SG poor areas, and later involving SG-rich parts, such as the face (19). Lipid analysis of the epidermis showed that the characteristic lipid barrier disruption in AD is a result of keratinocyte dysfunction and reduced levels of sebum lipids (20, 21). In contrast, PSO often starts on the scalp, especially in the early-onset form, and subsequently prefers sites with low sebum production, i.e. elbows and knees. However, in the distinct entity known as “sebopsoriasis” or “seborrhiasis” (seborrhoeic dermatitis + psoriasis), PSO lesions occur at the same sites as seborrhoeic dermatitis (22). This topographical coexistence, as well as other findings such as SG atrophy observed in the chronic phase of both diseases (23, 24), provide excellent starting points to further investigate the functional sebaceous (immuno)biology in PSO and AD (25, 26).

In this work, we aim to clarify the role of SGs in the development and disease homeostasis of AD and PSO. Therefore, we investigated and compared the spatial transcriptomic changes in SGs of lesional (L) and non-lesional (NL) human skin samples.

## Results

**Figure 1:**
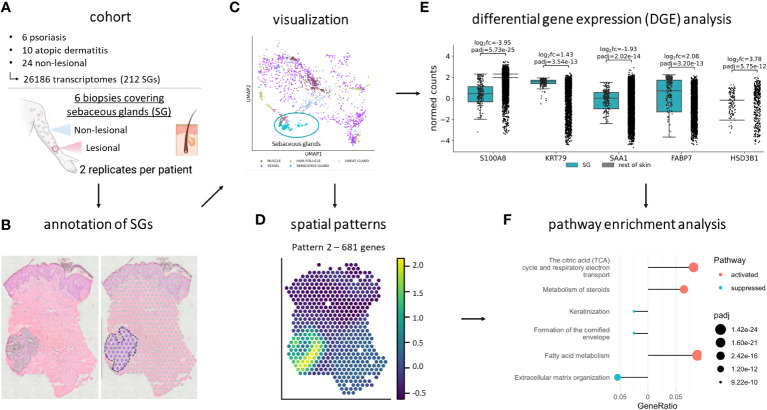
Study cohort and workflow. **(A)** The spatial transcriptomics dataset contains 6 lesional and non-lesional skin samples from psoriasis and atopic dermatitis patients. 6 psoriasis, 10 atopic dermatitis, and 24 non-lesional spots, containing 26,186 transcriptomes, of which 212 were of sebaceous glands, were analyzed. After **(B)** manual annotation for sebaceous glands and **(C)** visualization, the dataset was subject to **(D)** SpatialDE, **(E)** differential gene expression, and **(F)** pathway enrichment analysis. Created with BioRender.com.

SGs are characterized by their active lipid metabolism, lipid-related gene expression and protein abundance. Recently, sebocytes have been implicated in immunoregulatory functions (14). However, comprehensive analyses of their *in vivo* gene expression profile are lacking. Therefore, we aimed to identify differentially expressed (DEGs) and spatially variable genes (SVGs) in SGs of human NL, AD and PSO skin by spatial transcriptomics (Methods). Briefly, we manually annotated sebaceous glands in PSO, AD and NL skin samples (**Figures 1A, B**), visualized the data (**Figure 1C**), analyzed spatial patterns of SG-specific SVGs (**Figure 1D**), DEGs (**Figure 1E**) and pathway enrichments (**Figure 1F**).

### Sebaceous glands exert a specific pattern of gene expression in the skin

First, we identified the gene expression profile of SGs in NL skin samples. Our results showed that SGs have a specific gene expression signature that clearly distinguishes them from other structures within the skin (**Figure 1C**). Our analyses of SGs in NL skin compared to the rest of NL skin delivered a large set of 5,449 differentially expressed genes highlighting the unique characteristics of SGs (**Supplementary Table S2**, **Supplementary Figure S3**).

**Figure 2:**
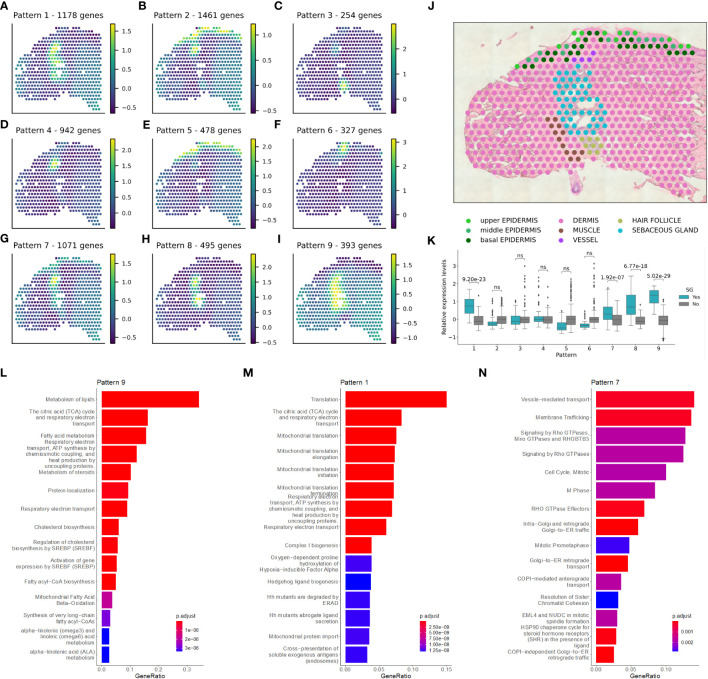
Sebaceous glands have a pivotal role in lipid metabolism-related tasks in non-lesional skin. Spatially variable genes and distinct spatial expression patterns were identified in non-lesional skin samples. **(A–I)** Enriched patterns for one replicate of a **(J)** non-lesional skin sample is shown. **(K)** Significant enrichment of sebaceous gland spots in the pattern intensities was calculated using Mann-Whitney U one-sided (greater) test. Pathway enrichment analysis in patterns **(L)** 9, **(M)** 1, and **(N)** 7.

To further dissect the spatial expression profile of SGs in NL skin, we identified SVGs and distinct spatial expression patterns (**Figures 2A–J**; Methods) (27). Four of the expression patterns were significantly enriched in SGs (**Figure 2K**): pattern 1 (1,178 genes, padj value: 9.20e-23), pattern 7 (1,071 genes, padj value: 1.92e-07), pattern 8 (495 genes, padj value: 6.77e-18), and pattern 9 (393 genes, padj value: 5.02e-29). Pathway enrichment analysis provided further insight into the SG-related patterns (**Supplementary Table S3**). Genes from pattern 9 revealed SG-typical pathways related to lipid, fatty acid, steroid, and cholesterol metabolism, and energy production (**Figure 2L**). Genes from pattern 1 were associated with mitochondrial function, the citric acid cycle and energy production (**Figure 2M**). Pattern 7 genes were linked to intracellular transport and cell cycle (**Figure 2N**).

### Sebaceous gland transcriptome is different in atopic dermatitis and psoriasis

**Figure 3:**
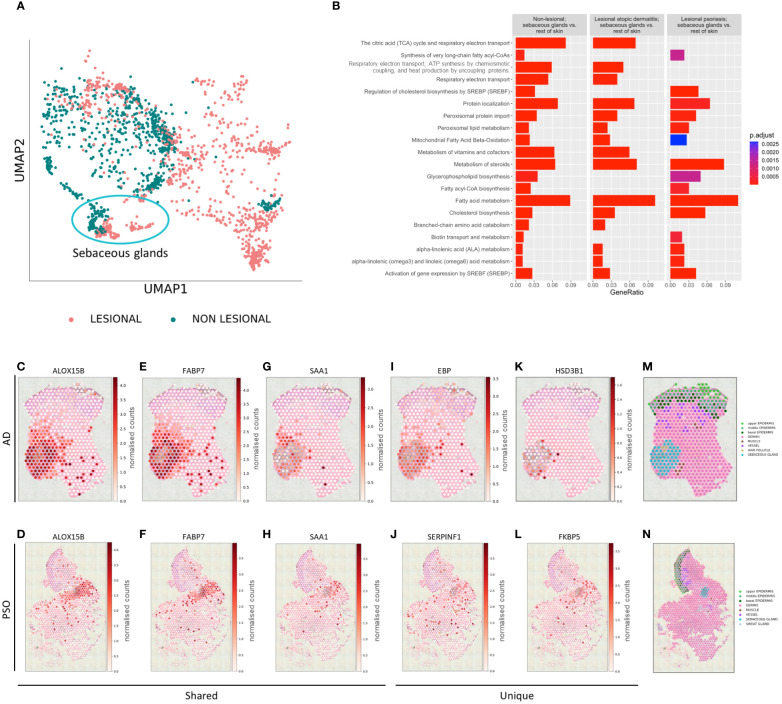
Sebaceous glands’ transcriptome changes in inflammatory microenvironment. **(A)** UMAP plot of gene expression of non-lesional and lesional SGs. **(B)** Top 20 Reactome pathways of enrichment analysis comparing non-lesional sebaceous glands vs. the rest of non-lesional skin and corresponding enrichment in lesional atopic dermatitis sebaceous glands vs. the rest of lesional atopic dermatitis skin, and lesional psoriasis sebaceous glands vs. the rest of lesional psoriasis skin. Selected spatial variable genes enriched in sebaceous glands **(C–H)** shared across atopic dermatitis and psoriasis, **(I, K)** unique to lesional atopic dermatitis and **(J, L)** unique to lesional psoriasis. Annotated lesional **(M)** atopic dermatitis and **(N)** psoriasis slides.

Extending our studies to L samples of AD and PSO, distinct gene expression profiles of NL and L SGs were revealed (**Figure 3A**). We identified genes with significantly altered expression levels in SGs compared to the rest of the skin in each of the above conditions and applied pathway enrichment analysis (**Figure 3B**). The top 20 pathways enriched in NL SGs compared to the rest of NL skin showed SG-typical functions related to lipid, cholesterol, or steroid metabolism, among others, and were used as a reference for the analysis of changes in DEGs in L SGs. Comparing the enriched pathways of DEGs in SGs in NL and AD skin, we found that SGs altered their specific gene expression signature related to synthesis of very long chain fatty acyl-CoAs, SREBP-regulated cholesterol biosynthesis, glycerophospholipid biosynthesis, and biotin transport in AD SGs. When assessing DEGs in SGs of PSO samples, pathways such as the citric cycle, electron transport and ATP synthesis, vitamin metabolism and branched-chain amino acid catabolism, which were enriched in NL and AD SGs, could not be identified. Importantly, gene clusters determining key SG functions such as peroxisomal lipid, steroid, fatty acid, cholesterol, and linoleic acid metabolism, as well as the activity of SREBP, were detectable in both AD and PSO.

To better understand the biology of SGs at a finer spatial scale, SVGs were identified using spatialDE (see also Materials & Methods). In both AD and PSO, SGs continued to express genes encoding key proteins for lipid metabolism and transport such as *ALOX15B* (**Figures 3C, D**), *APOC1, FABP7* (**Figures 3E, F**), *FADS1, FADS2, FASN, PPARG*, or *RARRES1* among others at high levels (**Supplementary Table S4**). Inflammation-related *SAA1* was also identified as a common AD/PSO SVG (**Figures 3G, H**). AD SG-specific SVGs included lipid metabolism-related genes such as *ACAD8, FADS6*, or *EBP* (**Figure 3I**), but also revealed inflammation-related *CCL17* and *HSD3B1* (**Figure 3K**). In PSO SGs, *SERPINF1* (**Figure 3J**) and immune function-related *FKBP5* (**Figure 3L**) were identified as SVGs. Other PSO-specific SVGs were the typical lipid metabolism-related gene *ACOT4, and S1PR3*, which is involved in proliferation and inflammation in PSO (28) (**Supplementary Table S4**). SVG expression was shown on previously annotated lesional atopic dermatitis (**Figure 3M**) and psoriasis (**Figure 3N**) slides.

### Sebaceous glands show profound changes in their lipid production-related gene expression profile in atopic dermatitis

**Figure 4:**
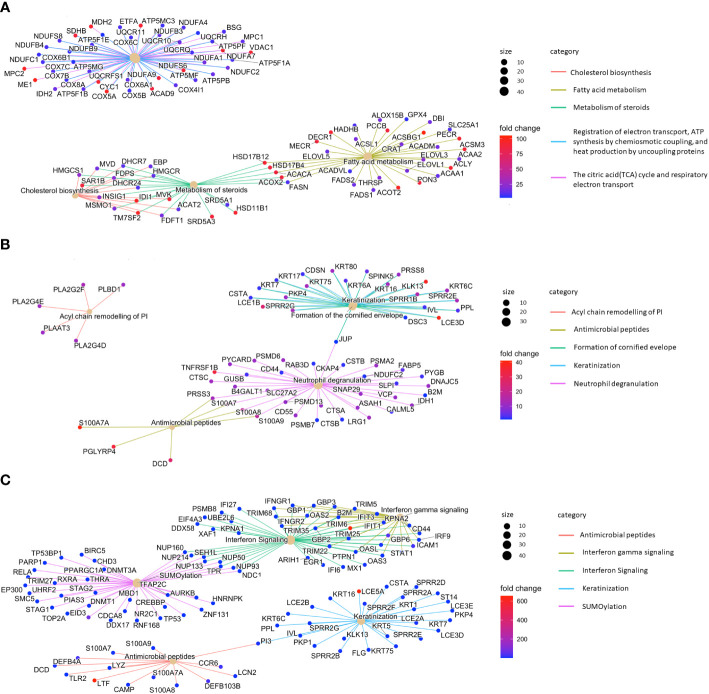
Sebaceous glands contribute to type 3 inflammation/Th17 immunity. Top 5 enriched pathways of **(A)** lesional atopic dermatitis sebaceous glands vs. non-lesional sebaceous glands, **(B)** lesional psoriasis sebaceous glands vs. non-lesional sebaceous glands, and **(C)** lesional psoriasis sebaceous glands vs. lesional atopic dermatitis sebaceous glands.

Having identified the genetic programs specific to SGs in the context of the whole skin, we aimed to define further disease-specific gene expression changes. Therefore, we compared the gene expression profiles of SGs in L AD skin with those of SGs in NL samples. The top 3 enriched pathways were cholesterol biosynthesis, fatty acid metabolism and steroid metabolism (**Figure 4A**). These results provide further evidence that SGs in AD actively modify their lipid profile already at the level of gene expression. Clusters such as ATP synthesis and electron transport further reveal an altered metabolic activity for SGs in AD skin.

### Gene signature encoding type 3/Th17-related immune functions distinguishes sebaceous glands in psoriasis and atopic dermatitis

By comparing the gene expression profile of SGs from L PSO and NL samples, we identified PSO-typical pathways related to differentiation (keratinization, cornified envelope formation) and inflammation (neutrophil degranulation, antimicrobial peptides; **Figure 4B**). In further analyses, we compared the gene expression profiles of L PSO vs. L AD SGs. In PSO, SGs gained immunocompetence. Besides immune features such as interferon signaling (e.g. *IFIT1/3, DDX58*) and production of antimicrobial peptides (e.g. *LTF, DEFB4A, S100A7-9*), significant differences were found in the expression of genes related to keratinization (e.g. *LCE5A, KRT5/7/16*) and SUMOylation in PSO (**Figure 4C**).

## Discussion

In this manuscript, we present an *in vivo* human spatial transcriptome signature analysis of SGs. Compared to the limitations of whole tissue analysis or *in vitro* data, spatial transcriptomics allowed us to define the transcriptome of sebocytes within small groups of cells *in vivo*. Using SpatialDE, a spatial gene clustering approach that enables expression-based tissue histology (27), we were able to study the biology of SGs at an even more granular scale.

SGs are well-defined, easily identifiable structures within the skin, composed predominantly of sebocytes. Although this minimizes annotation or contamination errors, a methodological limitation of our work is that the 55 µm spot size of the Visium Spatial Gene Expression slide (10x Genomics) used to analyze the samples does not allow conclusions to be drawn at the level of individual cells. This is more pronounced in acne samples, where the inflammatory cell infiltrate is also localized in the partially damaged pilosebaceous unit; therefore, we stuck to the two most common inflammatory skin conditions, PSO and AD, where the pilosebaceous unit is not the target of inflammation. A comparison of our data to the SG-specific transcriptome of acne lesions would have been desirable. Nevertheless, aside from the above mentioned limitations, published whole tissue analyses do not provide sebocyte-specific gene expression data (29), while available single cell RNA results on acne samples lack sebocyte-specific data (30). Future spatial transcriptomics studies focusing on SGs in acne lesions will allow further conclusions on the specific role and comparison of SGs in acne and other inflammatory skin diseases. Other limitations are that SGs are rare in lesional PSO and AD samples, and the size of the cohort analyzed in our study is also small, although the total of more than 26,000 transcriptomes analyzed allowed us to delve deep into the SG transcriptome.

While confirming the overexpression of lipid metabolism-related genes in SGs, our spatial transcriptomics analysis shed light on previously unstudied pathways. The highly active cell type-specific lipid metabolism of sebocytes has been progressively revealed over the last two decades of sebocyte research (14). Here, we confirm the *in vivo* relevance of widely studied enzymes and signaling pathways like delta-6 desaturase/FADS2 or stearoyl-coenzyme A desaturase (31, 32). Furthermore, the previously reported central role of nuclear receptors such as PPARs or retinoic acid (33–35), and the characterization of other transcription factors such as SREBP-1 or FoxO1 (36) in the regulation of SG proliferation and lipid metabolism (37) are supported by our findings. Based on our data, linoleic acid, a known activator of PPAR-γ and also the source of arachidonic acid, could be a potent natural stimulus behind the unique features of sebocytes (38, 39). We also confirmed the central role of genes involved in lipid synthesis (*FASN, THRSP*, and *ELOVL5*), metabolism (*FADS2* and *ACSBG1*) and transport (*APOC1*), and keratinization (*KRT79*), which were found to be expressed in a combined subpopulation of healthy, L and NL AD inner root sheath and SG cells (40). In the present study, we identified each one of these genes and many more as SVGs in L AD and PSO SGs. In addition, our transcriptome analyses revealed enzymes and pathways for further studies, such as the role of SUMOylation and the HSP90 chaperone cycle for steroid hormone receptors in sebocytes.

The results of our study support the postulated inflammatory capacity of sebocytes in AD. AD is characterized by dry skin and inflammation, which is primarily associated with an impaired skin barrier. The findings that AD skin has low levels of sebocyte-specific lipids (20, 41, 42), and a recent publication showing that the amount of sebum secreted by SGs was decreased in AD patients and was negatively correlated with barrier function and disease severity (43), further support that SGs may play an active role in the pathogenesis of AD. Importantly, a recent study has also linked the cytokine milieu of AD to sebocyte functions by showing that IL-4 upregulates the expression of 3β-hydroxysteroid dehydrogenase 1 (HSD3B1), a key enzyme in the conversion of cholesterol to sebum lipids (44). Here, we support these findings by identifying *HSD3B1* as an AD SG-specific SVG.

SGs appear to be involved in type 2/Th2 inflammation. *ALOX15B*, a common AD/PSO SVG, is a key player in fatty acid metabolism, and cholesterol homeostasis. In our previous studies investigating the eicosanoid/docosanoid signaling in the skin of human AD patients, we found that the sum of 15-LOX metabolites was significantly increased (45). Furthermore, studies have shown that in activated human macrophages, *ALOX15B* is induced by the Th2 cytokines IL-4 and IL-13 and has an effect on IL-4-induced *CCL17* in an *SREBP-2*-dependent manner (46). This further supports a potential involvement of SGs in type 2/Th2-inflammation. However, the identification of *ALOX15B* as an SVG in PSO SGs requires further validation to define its role in type 3/Th17-inflammation.

We found further evidence for the active contribution of SGs in inflammation. *CCL17* plays a potential role in the pathogenesis of AD (47), which was also identified as an AD-specific SVG in the present study. While *SAA1* encoding serum amyloid A1, previously described as a marker of TLR 1/2- and 4-activated SGs (8), was also found to be a common SVG of AD/PSO SGs in the present work, highlighting the importance of further investigating the inflammatory capacity of SGs.

An alteration of the retinoic acid signaling at the level of the SGs may be pathologically relevant, as *RARRES1* expression levels were also altered in SGs of AD and PSO samples. Notably, *RARRES1* is one of the key genes found to be upregulated in skin samples from acne patients treated with the potent skin drying agent isotretinoin, as well as in both the SEB1 (48) and SZ95 sebocyte cell lines (49) in response to isotretinoin.

Overall, the SG transcriptome signature in AD revealed numerous genes involved in the formation of the lipid skin barrier. The clusters of mitochondrial functions, ATP synthesis and respiratory electron transport that were altered in AD SGs provide further important starting points for studies on how changes in lipid production might be linked to an altered energy expenditure (50, 51).

Our data confirmed that PSO SGs not only maintained their active lipid metabolism, but also acquired immune-competence via their gene expression profile. PSO is characterized by atrophy and sometimes absence of SGs in the affected skin samples, raising the questions of whether this plays a role in the development and progression of the disease and whether the alterations in the expression of lipid metabolism-related genes (*AWAT2, DHCR7, ELOVL5* or *FAR2*) identified in this study are specific to PSO. The involvement of PSO SGs in skin inflammation was confirmed by comparing SGs from PSO samples with SGs from NL and AD samples. The detected transcripts encoding keratins and differentially down-regulated genes related to cell cycle and proliferation suggest that the driving mechanism behind SG atrophy may share similarities, such as the involvement of NOTCH signaling, but is generally different in the two diseases. Immune-related clusters, such as interferon signaling, neutrophil activation and the induction of genes encoding antimicrobial peptides, clearly dissected the two diseases also at the level of SGs, suggesting an active contribution of SGs to type 3/Th17 inflammation.

Notably, *S1PR3* was identified as a PSO SG-specific SVG in our study, suggesting an involvement of SGs in the pathogenesis of PSO. The lncRNA H19/miR-766-3p/S1PR3 axis has previously been shown to contribute to keratinocyte hyperproliferation and skin inflammation in PSO via the AKT/mTOR pathway (28). The PSO-specific SVG *SERPINF1* may also play a role in the immune regulation of PSO (52).

*FKBP5* was identified as another PSO-specific SVG. Recently, the immunoregulatory *FKBP5* has been shown to contribute to NF-κB-driven inflammation and cardiovascular risk (53), and is also associated with depression susceptibility (54, 55). Both cardiovascular risk and depression are known and common comorbidities of psoriasis (56, 57). Further studies are needed to investigate a potential role of *FKBP5* in the link between systemic inflammation, cardiovascular risk and depression susceptibility in psoriasis patients.

In conclusion, this study provides human *in vivo* data which confirmed that beyond altering their lipid metabolism in a disease-specific manner in an inflammatory microenvironment, SGs can be considered as an active and immunocompetent structure in L skin with possible pathological and therapeutic relevance. Moreover, our data serve as a starting point for further studies at protein level to better understand the role of SGs in inflammatory skin diseases in the future.

## Materials & methods

### Study cohort and spatial transcriptomics

The study cohort leverages patients from the Schäbitz et al. study (58). L and NL skin from each patient was collected and subsequently processed using the software SpaceRanger-1.0.0 from 10x Genomics. L skin was defined by clinical presence of typical hallmarks of AD or PSO inflammation, such as involvement of predilection sites, erythematous papules and plaques, or scaling. After taking the biopsies, the diagnosis was confirmed by 2 independent dermatopathologists, considering typical histological hallmarks of AD or PSO, including presence of immune cells, spongiosis, acanthosis, papillomatosis, and hyperkeratosis, amongst others. NL skin was defined as skin clinically and histologically absent of the mentioned AD and PSO (or any other dermatosis) hallmarks. The study was approved by the local ethics committee (Klinikum Rechts der Isar, 44/16 S). Each patient gave written informed consent for sample collection for research purposes.

### Spatial transcriptomics data preprocessing

Leveraging the cohort from Schäbitz et al. (58), we performed the preprocessing using `scanpy` (59). First, we conducted quality control on spot and gene level. Spots having a mitochondrial fraction above 25%, less than 30 genes, and less than 500 UMI-counts or more than 500,000 UMI-counts were filtered out. Genes were required to be measured in at least 20 spots. The R-package `scran` (60) was used to normalize the data using size factors. We added a pseudo count of 1 to the normed counts and transformed them into log counts per million (logCPM). Next, we identified highly variable genes for each specimen using the flavor cell_ranger. We corrected for technical artifacts caused by the project co-variate using `scanorama` (61). In order to embed the data in 2D, we calculated principal components (PCs) and selected n_pcs = 15 explaining the most variance. PCs were leveraged to create a nearest neighbor graph using the default parameters. Using the graph, the data was embedded in 2D using UMAP (62). For the downstream analysis we selected only those specimen having SG annotations. In total we got 1 PSO, 1 AD, and 1 non-lesional sample with 2 replicates each (6 slides in total) (**Supplementary Table S1**).

### Differential gene expression and pathway enrichment analysis of spatial transcriptomics

To identify significantly up- and down-regulated genes in SGs at a spatial resolution, we compared spots annotated as SG with the remaining spots using the R-package ‘glmGamPoi’ (63). Raw counts and size factors which have been calculated during the preprocessing step were used as input for the differential gene expression (DGE) analysis. In addition, we also considered biological variances, i.e., cellular detection rate (cdr), patient heterogeneity, and tissue layers. Variables of the differential gene expression (DGE) analysis were NL skin, AD, PSO, and a pool of PSO and AD. The following designs were used.


$$Y_{s,ɡ\;}∼\;cdr\;+\;patient\;+\;annotation\;+\;condition$$

and


$$Y_{s,ɡ}∼cdr\;+\;annotation\;+\;condition$$

Here, $Y_{s,ɡ}$ is the raw count of gene ɡ in a spot s . The later design was used to compare L, PSO vs. AD in **Figure 4C**, as the design matrix needed to be of full rank. P-values were corrected using the multiple testing method of Benjamini-Hochberg (BH) (64). In addition, DEx genes had to have a adj. p−value ≤ 0.1 and $|lo{ɡ}_{2}FC|>1$ .

Pathway enrichment analysis was performed using the Bioconductor packages ‘ReactomePA’ (65) and ‘org.Hs.eg.db’ (66). Pathways were considered enriched at a false discovery rate (FDR) of 10%, corrected with BH.

### Discovering spatial patterns and variable genes

We used spatialDE (27), which allowed us to determine spatial patterns and their associated genes per sample. Following the spatialDE workflow, we assumed normal distributed data, corrected for library size and ran spatialDE with default settings to obtain spatial variable genes (SVGs). Automatic expression histology (AEH) was used to identify spatial patterns using the previously observed and prefiltered SVGs requiring a q-value < 0.05. We set the number of expected patterns *C* to nine and used the mean length scale as optimal characteristic length scale parameter *l* as recommended by spatialDE. In order to determine whether a pattern is enriched in a SG, we used the alternative hypothesis that pattern intensity in SG is greater than in other spots. The tests for all patterns on a specimen were conducted using the one-sided Mann-Whitney U test (67) in the python package `statannotations` (68). P-values were corrected with the multiple test correction method Bonferroni (69). We called the null hypothesis rejected if the adj. p−value ≤ 0.05 . Default parameters of Bioconductor’s R package “ReactomePA” were used for p-value and q-value cut-offs, and a minimal gene set size of five was required.

## Data availability statement

The datasets presented in this study can be found in online repositories. The names of the repository/repositories and accession number(s) can be found below: https://www.ncbi.nlm.nih.gov/, GSE206391. Source code is available at github: https://github.com/MendenLab/ST_SebaceousGlands.

## Ethics statement

The studies involving humans were approved by Klinikum Rechts der Isar, Munich, Germany, 44/16 S. The studies were conducted in accordance with the local legislation and institutional requirements. The human samples used in this study were primarily isolated as part of our previous study (58) for which ethical approval had been obtained. Written informed consent for participation was not required from the participants or the participants’ legal guardians/next of kin in accordance with the national legislation and institutional requirements.

## Author contributions

PS: Data curation, Formal analysis, Investigation, Methodology, Writing – original draft, Writing – review & editing. CH: Conceptualization, Data curation, Formal analysis, Investigation, Methodology, Software, Validation, Visualization, Writing – review & editing. ASc: Data curation, Investigation, Writing – review & editing. MJ: Data curation, Investigation, Writing – review & editing. AP: Investigation, Writing – review & editing. SE: Conceptualization, Methodology, Resources, Writing – review & editing. ASz: Formal analysis, Writing – review & editing. MS: Formal analysis, Writing – review & editing. FG: Formal analysis, Writing – review & editing. CZ: Formal analysis, Writing – review & editing. TB: Resources, Supervision, Writing – review & editing. MM: Conceptualization, Data curation, Investigation, Methodology, Resources, Supervision, Visualization, Writing – review & editing. KE: Conceptualization, Data curation, Funding acquisition, Methodology, Resources, Supervision, Writing – review & editing. DT: Conceptualization, Data curation, Methodology, Resources, Supervision, Writing – original draft, Writing – review & editing.

## Supplementary material

Supplementary Table 1 Study cohort.

Supplementary Table 2 Differentially expressed genes of non lesional sebaceous glands vs. the rest of non-lesional skin.

Supplementary Table 3 Pathway enrichment analysis of patterns where sebaceous glands were significantly enriched in non-lesional skin (patterns 1, 7, 8, and 9).

Supplementary Table 4 Unique and shared spatially variable genes of atopic dermatitis and psoriasis sebaceous glands.
