# Supplementary figures and images for: Spatial transcriptomics reveals altered lipid metabolism and inflammation-related gene expression of sebaceous glands in psoriasis and atopic dermatitis
Source: Front Immunol. 2024 Feb 16;15:1334844. doi: 10.3389/fimmu.2024.1334844 (PMC10904577; doi:10.3389/fimmu.2024.1334844)

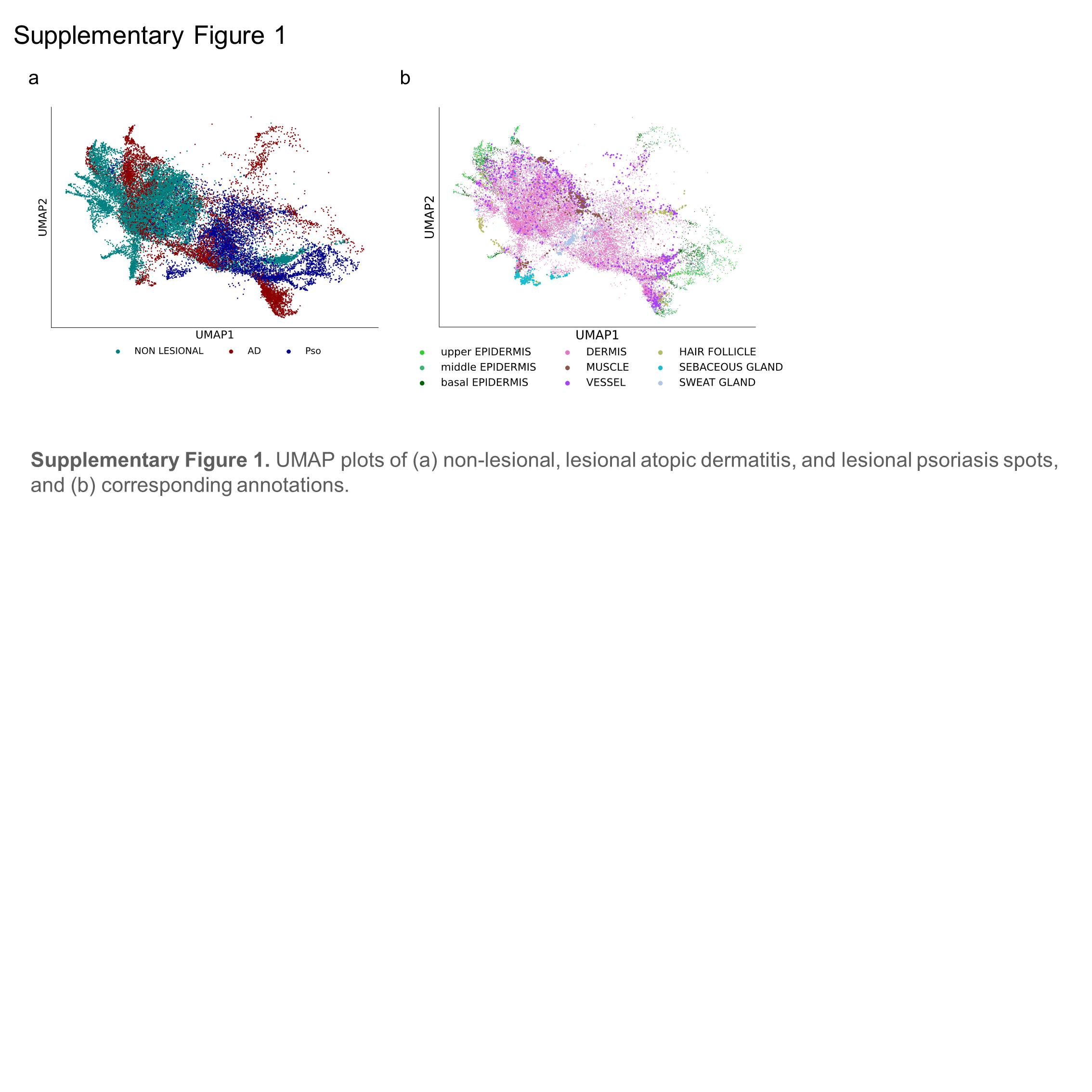

Supplement: Supplementary file 1 [file Image_1.tif]

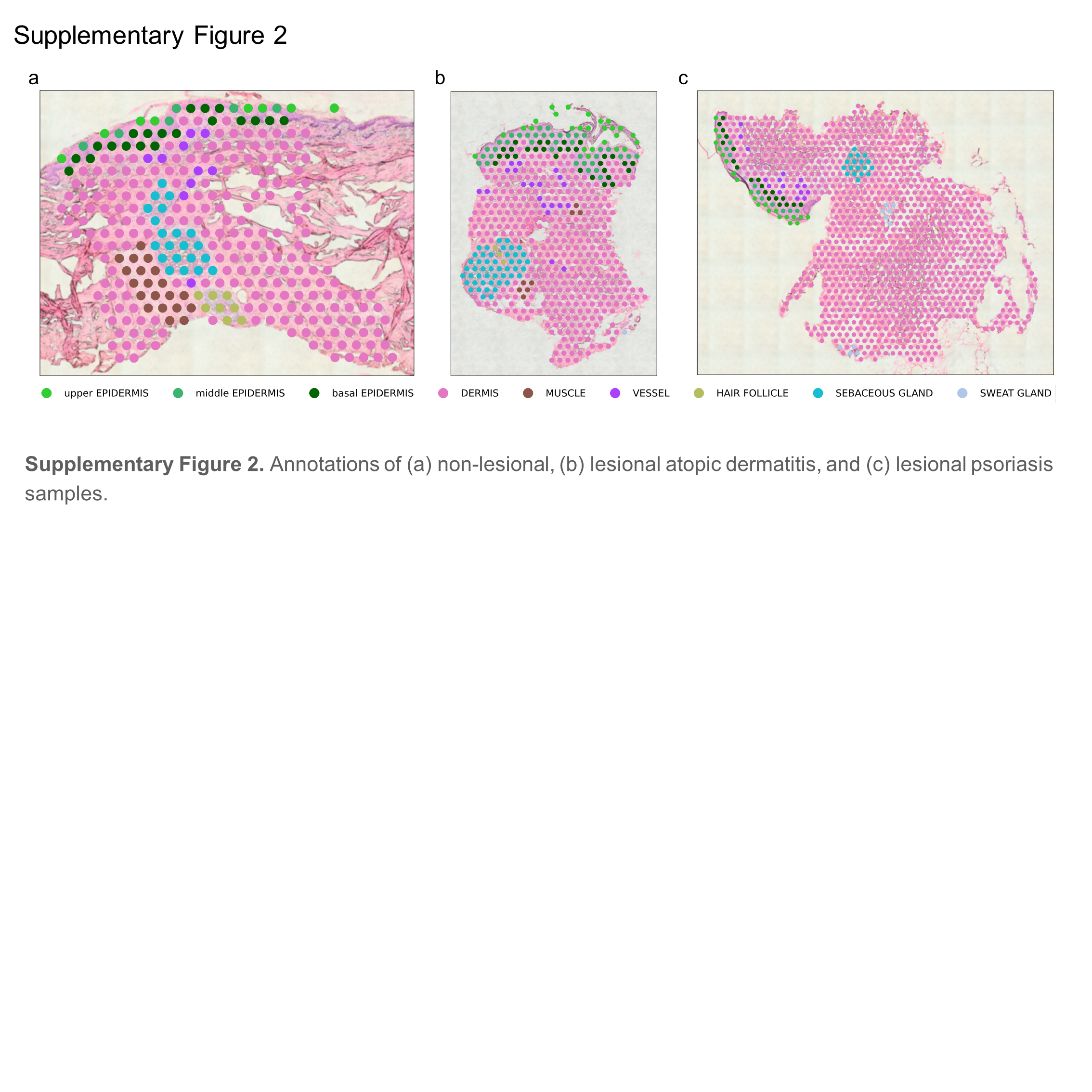

Supplement: Supplementary file 2 [file Image_2.tif]

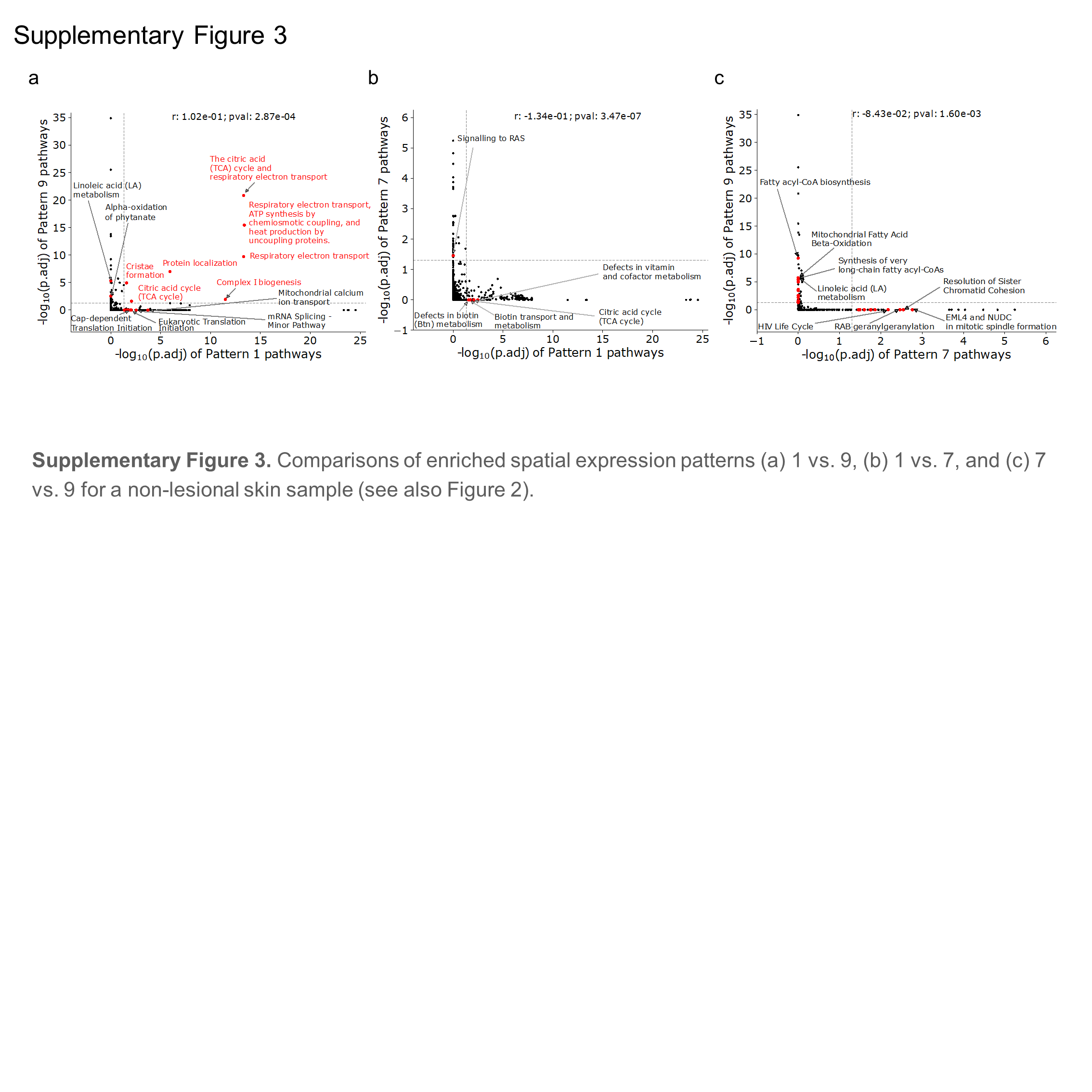

Supplement: Supplementary file 3 [file Image_3.tif]

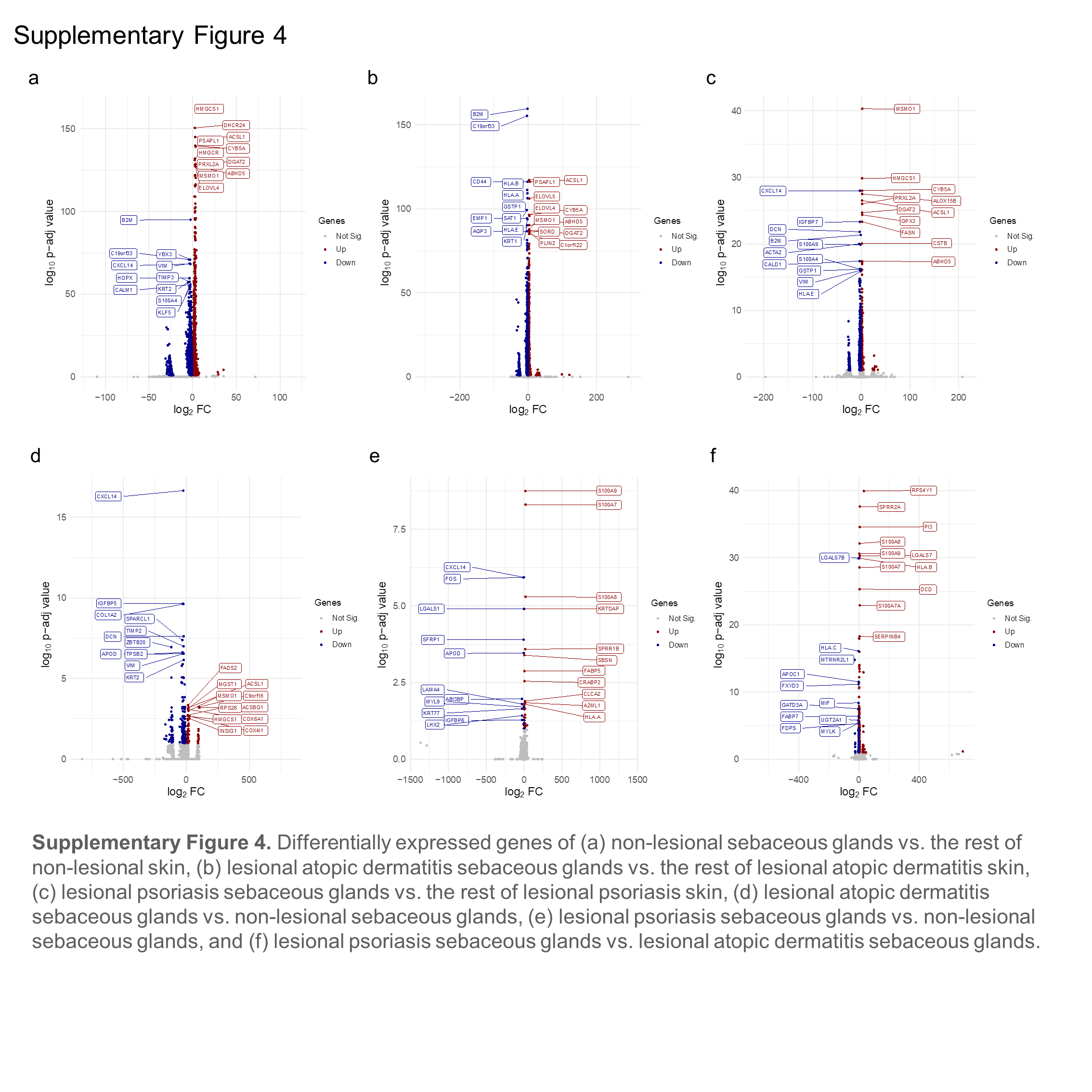

Supplement: Supplementary file 4 [file Image_4.tif]
